# Synthesis of 1,2,3-Triazolo[4,5-*h*]quinolone Derivatives with Novel Anti-Microbial Properties against Metronidazole Resistant *Helicobacter pylori*

**DOI:** 10.3390/molecules22050841

**Published:** 2017-05-20

**Authors:** Mohammad Abu-Sini, Amal Mayyas, Nehaya Al-Karablieh, Rula Darwish, Yusuf Al-Hiari, Talal Aburjai, Shereen Arabiyat, Luay Abu-Qatouseh

**Affiliations:** 1 Faculty of Pharmacy, Al-Zaytoonah University of Jordan, Amman 11733, Jordan; mohammad.abusini@zuj.edu.jo; 2 Faculty of Health Sciences, American University of Madaba, Madaba 11821, Jordan; a.mayyas@aum.edu.jo; 3 Hamdi Mango Center for Scientific Research, University of Jordan, Amman 11914, Jordan; n.alkarablieh@ju.edu.jo; 4 Faculty of Pharmacy, University of Jordan, Amman 11914, Jordan; rulad@ju.edu.jo (R.D.); hiary@ju.edu.jo (Y.A.-H.); aburjai@ju.edu.jo (T.A.); 5 Salt College, Al-Balqa Applied University, As-Salt 19117, Jordan; Shereen@bau.edu.jo; 6 Faculty of Pharmacy, University of Petra, Amman 11914, Jordan

**Keywords:** *H. pylori*, triazoloquinolone derivatives, synergism

## Abstract

*Helicobacter pylori* infection can lead to gastritis, peptic ulcer, and the development of mucosa associated lymphoid tissue (MALT) lymphoma. Treatment and eradication of *H. pylori* infection can prevent relapse and accelerate the healing of gastric and duodenal ulcers as well as regression of malignancy. Due to the increasing emergence of antibiotic resistance among clinical isolates of *H. pylori*, alternative approaches using newly discovered antimicrobial agents in combination with the standard antibiotic regimens for the treatment of *H. pylori* are of major importance. The purpose of the present study was to investigate the effect of newly synthesized 8-amino 7-substituted fluoroquinolone and their correspondent cyclized triazolo derivatives when either alone or combined with metronidazole against metronidazole-resistant *H. pylori*. Based on standard antimicrobial susceptibility testing methods and checkerboard titration assay, all of the tested compounds showed interesting antimicrobial activity against 12 clinical strains of *H. pylori*, with best in vitro effect for compounds **4b** and **4c**. Fractional inhibitory concentration (FIC) mean values showed synergistic pattern in all compounds of Group 5. In addition, additive activities of some of the tested compounds of Group 4 were observed when combined with metronidazole. In contrast, the tested compounds showed no significant urease inhibition activity. These results support the potential of new fluoroquinolone derivatives to be useful in combination with anti-*H. pylori* drugs in the management of *H. pylori*-associated diseases.

## 1. Introduction

*H. pylori* is a gram negative, spiral shaped, microaerophilic bacterium [1,2]. Due to its unique growth and virulence characteristics, such as production of as urease and CagA and VacA toxins, *H. pylori* has been recently recognized as a major causative agent of different diseases such as chronic gastritis, peptic ulcer, gastric adenocarcinoma, and gastric mucosa-associated lymphoid tissue (MALToma) [3,4,5,6]. Eradication of *H. pylori* has been shown to result in ulcer healing, prevent peptic ulcer recurrence, and reduce the prevalence of gastric cancer in high risk populations [7,8]. Treatment of *H. pylori* infections is routinely achieved by use of at least two antibiotics, especially amoxacillin and clarithromycin or metronidazole, and a proton pump inhibitor (triple and more recently quadruple therapy) [9,10,11]. Although the conventional treatment for eradication therapy of *H. pylori* allows obtaining high cure rates, the eradication failure rate remains of 5–20% [12]. This can be explained by lack of patient compliance with the drug regimens and the development of antibiotic resistance [13,14,15]. Therefore, a search for new approaches with high efficacy and safety against *H. pylori* infection is necessary.

Fluoroquinolones have gained extensive use owing to their favorable pharmacokinetics and broad antimicrobial spectra [16]. Various studies have reported that fluoroquinolones have many interesting biological activities like analgesic [17], antidiabetic [18], antibacterial [19,20], and antitumor activities [21,22]. The widespread use of fluoroquinolones has had its consequences, as selective pressure has increased resistance for some species over the years [23]. So far, low level of resistance against fluoroquinolones among *H. pylori* clinical strains is reported [24,25]. In a previous work, the biological activities of a group of 8-nitro 7-substituted fluoroquinolone derivatives have been evaluated and strong antibacterial activity against *Escherichia coli* and *Staphylococcus aureus* was observed [26,27]. In general, the synthesis of novel 8-nitro 7-substituted fluoroquinolone derivatives was evaluated for biological activity. In the present study, the antibacterial activity of previously described 8-nitro 7-substituted fluoroquinolone derivatives [28] and two newly synthesized 8-nitro 7-substituted fluoroquinolone derivatives was tested against twelve clinical strains and one control strain of *H. pylori* which are resistant to metronidazole. In addition, the effect of the potentially effective derivatives in combination with metronidazole against metronidazole resistant *H. pylori* strains was evaluated.

## 2. Results and Discussion

### 2.1. Synthesis of New Compounds

**Scheme 1:**
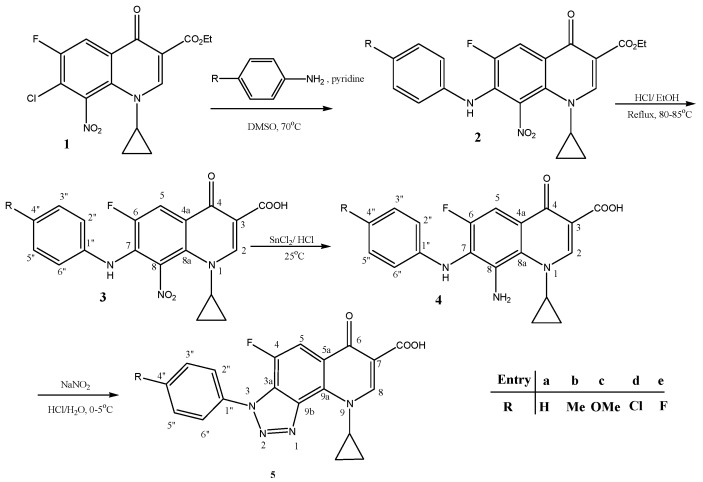
General procedure for the synthesis of novel target compounds **4**, **5** (**a**–**e**). DMSO: dimethylsulfoxide.

Synthesis of esters 1, compounds **2**, **3** (**a**, **d**, **e**) were conceded following a previously reported procedure with good yields [27,29]. Compound **5c** was prepared as previously described [30]. Due to the novelty and remarkable activity of **4b**, **5b**, and **4c**, we report these compounds for the first time in this work. All compounds **2**–**5** (**a**, **d**, **e**) were prepared in this work for biological activity, and their spectral data was reported previously [29,31,32]. Upon coupling the substituted aniline to cipro ester 1, compounds **2a**–**e** were synthesized, (Scheme 1).

This step was followed by a tedious hydrolysis process of nitro ester 2 to generated nitro acid targets 3. Reduction of nitro acids **3a**–**e** using stannous chloride in acidic media yielded the 8-amino acid derivatives **4a**–**e** with reasonable yield. These targets were identified and characterized by infrared spectroscopy (IR), mass spectrometry (MS), and ^1^H- and ^13^C-NMR spectroscopic analyses. The spectral data of **4**, **5** (**b**, **c**) are presented in the experimental part since they are most active and novel.

The proton ^1^H-NMR spectra of the reduced compounds (**4a**–**e**) contained a doublet for H-5 (^3^*J*_H-F_ = 10–13 Hz) at ~8.0 ppm due to coupling with fluorine atom at C-6. A singlet peak for H-2 at ~8–9.0 ppm established that the fluoroquinolone system 4 is there. Both methyl and methoxy have shown singlets in the range of 2.3–3.7 in the ^1^H-NMR spectra. The aromatic protons peaks were present in the ranges of 6.8–7.2 which confirm the presence of the aniline side chain. Furthermore, the appearance of a broad singlet stands for 2 protons at 4.2–5.5 ppm indicated that the reduction step had proceeded successfully to give compounds **4b** and **4c**. The disappearance of such broad singlet confirmed that compounds **4b** and **4c** had undergone the diazotization and further cyclization reactions to give compounds **5b** and **5c**, respectively.

For ^13^C spectra, all carbon peaks of **4** and **5** were detectable by their number, position and orientation in the charts. These signals confirmed that the aryl amine chain is fused in compounds **4**, **5** (**a**–**e**). For the fluoroquinolone part, the ^13^C-NMR spectra of compound **4** contained a doublet (^1^*J*_C-F_ = 250 Hz) at ~150 ppm for C-6 (C4 for compound **5**); which indicated the presence of the fluoroquinolone nucleus in all of these compounds. This was also confirmed from depth analysis. The splitting of the neighboring carbon signals of 4 at C-5 and C-7 (**5** and **3a** in compound **5**) into doublet peaks in these compounds (^2^*J*_C-F_ ~ 20 Hz) effectively confirmed that they were all vicinal to a fluorine atom. Appearance of two symmetrical long signals in depth confirmed the aromatic side chain presence.

### 2.2. Antimicrobial Activity against Metronidazole-Resistant H. pylori

Previous work on the antimicrobial activity of different quinolones derivatives reported significant variation in the susceptibility of the tested compounds against Gram positive and Gram negative bacteria [33,34]. This was relatively ascribed to the hydrophobic/hydrophilic properties of their structures, with no specific pattern [27,28,29]. *H. pylori* clinical isolates exhibit unique biological characteristics among all eubacteria. Being a microaerophilic slow growing microorganism would provide a plasticity of its response toward the antimicrobial agents particularly in vivo. This study is an attempt toward developing putative novel anti-*H. pylori* agents derived from 8-amino 7-substituted and their correspondent cyclized triazoloquinolone derivatives. The results of initial screening showed that all of the tested compounds have antimicrobial activity against *H. pylori*, with remarkable potent effects of compound **4b**, followed by compounds **4a** and **4c**, respectively (Table 1). Compounds of Group 5 (7–8 triazole) showed good but lower activity compared to compounds of group 4. The MIC values of the tested compounds against the clinical strains were in the range of 0.6–5 μg/mL which are comparable to ciprofloxacin. Compounds **4a**–**e** and **5b**–**e** exhibited significant in vitro anti-*H. pylori* activity (Table 2). The highest antibacterial activity (lowest MIC values) was reported for compound **4b**, followed by compounds **4a** and **4c**, respectively. It is worth mentioning that our compounds are more active than metronidazole on the resistant strains. This activity might be linked to their lipophilicity [26,27].

In terms of the antimicrobial effects of the combinations of the tested compounds with metronidazole against the metronidazole resistant strains, this work reported that Group **5b**–**e** developed synergistic pattern in all its compounds with fractional inhibitory concentration (FIC) indices range of 0.283–0.349. Surprisingly, our results showed that all tested compounds of Group 4 lost synergistic activity with metronidazole combination and showed mainly indifferent activity of metronidazole **4a**, metronidazole **4b**, and metronidazole **4c** combination with fractional inhibitory concentration (FIC) indices of 1.025, 1.192, and 2.212, respectively (Table 3), although these compounds when tested alone showed high activity against the tested strains. It could be suggested that C-8 amino group of these compounds was responsible for such phenomenon through physical interaction with metronidazole. Such interaction might be attributed to intermolecular hydrogen bond interaction of C-8 primary amine in these compounds with metronidazole.

Moreover, combination of metronidazole + **4d** and metronidazole + **4e** showed additive effect, with FIC indices of 0.692 and 0.718, respectively (Table 3). The combination of metronidazole + **4c** showed antagonistic effect, with FIC indices of 4.078 when tested against strain 11. The Most active compounds **4a**, **4b**, and **4c**, suffered significant loss in antibacterial activity due to significant hydrogen bond interaction possibly with both N-H bond at 8-amino leading to loss polar characters. On the other hand, less active derivatives **4d** and **4e** showed minor change in FIC values, due to weak original antibacterial activity. Group 5 showed best FIC with synergistic mode in all derivatives since they were unable to form such intermolecular hydrogen bond and thus no effect on final lipophilicity and consequently on net activity.

### 2.3. Inhibitory Effects of the Synthesized Compounds against H. pylori Urease Enzyme

*H. pylori* infection may be controlled by reduction of its pathogenicity through targeting its virulence factors [35]. The most important virulence factor of *H. pylori* is the urease enzyme, which is essential for the optimal colonization and survival of this pathogen in the acidic conditions of the human stomach [35]. In this work, 8-amino 7-substituted and their correspondent cyclized triazoloquinolone derivatives were evaluated for their potential inhibitory effect against *H. pylori* urease. The compounds were tested for their inhibitory effects in the range of 0.5–1000 μg/mL by the standard colorimetric method described in the materials and methods section, and the compounds that showed less than 60% inhibition were considered to have no significant effect [36]. Our study showed no significant urease inhibitory effect for compounds **4a**, **4b**, and **4c** at 1000 μg/mL with inhibitory percent 13, 10, and 33%, respectively. The other compounds were found to be inactive at the specified concentrations.

### 2.4. Structural Activity Relationship Studies

The work discloses that both series of **4** and **5** (**a**–**e**) exhibited significant inhibitory activity against *H. pylor*i. The inhibitory activity of the reduced 8-amino derivatives **4** (**a**–**d**) were much stronger than the triazoloqhinolones **5** (**b**–**e**). Compounds **4b** and **4c** exhibited comparable MIC (μg/mL) compared to ciprofloxacin against some clinical strains (Table 2). These data reveal that series **4** can open the door for strong inhibitors against *H. pylori*.

In previous work [26,27,28,29], we established that the activity of these FQs mendacities mainly within the 4-oxo-pyridine-3-carboxylic acid system present in all compounds prepared (**3**–**5**). Losing the free COOH group in compounds **2**–**5** reduced the inhibitory activity against *H. pylori* significantly [29]. Furthermore, it was noted that FQs lack any aniline substituent at C7 as in compounds **3**, **4** (C3 in **5**) showed no or low activity against *H. pylori*. This conclusion entitles that increasing lipophilicity through aniline substituents at C-7 can significantly improve the activity against *H. pylori*. Evidently, most of our derivatives are lipophilic compound and have shown the reasonable inhibitory activity against *H. pylori* [29]. These findings were emphasized previously by our group: highly lipophilic fluoroquinolone ligands improve antibacterial activity against Gram positive bacteria in particular, and to some extent against Gram negative bacteria [26,27,28]. Within the same vein, our group has emphasized that Gram negative activity was boosted through C-7 and C-8 hydrogen bond network of fluoroquinolone systems as represent by compounds of series **4**. The activity of compounds **4a**–**e** against Gram negative *H. pylori* could also be attributed also to the extra hydrogen bond network that might be formed on C-7 (NH) and C-8 (NH_2_) groups in our FQs. The extraordinary activity of compound **4b** in particular against *H. pylori* can be explained by high lipophilicity of the methyl substituent and H-B network enforced by NH_2_ group. Most possible that extra H-B imposed by the 8-amino group can leads to intermolecular hydrogen bond with the combined drug metronidazole and hide the active group in both FQ and metronidazole from acting on their target in *H. pylori*. This interaction between highly polar groups in both compounds might explain the low activity of the combination against *H. pylori*. The cyclized triazoloquinolones of series **5** displayed lower inhibitory activity possibly due to loss of free H-B amino group.

## 3. Materials and Methods

### 3.1. Materials and Instruments

All chemicals, reagents, and solvents were of analytical grade and used directly without further purification. *p-*Anisidine and *p*-toluidine were purchased from Fluka (Buchs, Switzerland). The reducing agent, anhydrous stannous chloride crystals, was purchased from Fluka (Buchs, Switzerland). Sodium nitrate was purchased from Sigma Aldrich (St. Louis, MO, USA). Melting points (m.p.) were determined in open capillaries on a Stuart scientific electro-thermal melting point apparatus (Stuart, Staffordshire, UK) and are uncorrected. Thin layer chromatography (TLC) was performed on 10 × 10 cm^2^ aluminum plates pre-coated with fluorescent silica gel GF254 (ALBET, Berlin, Germany) and was visualized using UV lamp (at 254 nm wave length/short wave length/long wavelength). Mobile phase mixtures were 94:5:1 chloroform–methanol–formic acid (CHCl_3_–MeOH–FA) (system 1) and 50:50 (*n*-hexane–ethyl acetate) (system 2). Nuclear magnetic resonance spectra were recorded on a Varian Oxford-300 (300 MHz) spectrometer and a Bruker, Avance DPX-300 spectrometer, a Bruker Avance-400 (400 MHz) Ultrashield spectrometer, and on Bruker 500 MHz-Avance III (500 MHz) (Bruker, Billerica, MA, USA). Deuterated dimethylsulfoxide (DMSO-*d*_6_) and deuterated chloroform (CDCl_3_) were used as solvents in sample preparation, unless otherwise indicated. The chemical shifts were reported in ppm relative to tetramethylsilane (TMS), which was used as an internal reference standard. ^1^H-NMR data are reported as follows: chemical shift (ppm), (multiplicity, coupling constant (Hz), number of protons, the corresponding proton(s). Infrared (IR) spectra were recorded using Shimadzu 8400F FT-IR spectrophotometer (Shimadzu, Kyoto, Japan). The samples were prepared as potassium bromide (KBr) (Sigma, St. Louis, MO, USA) disks. High-resolution mass spectra (HRMS) were measured in positive ion mode using electrospray ionization (ESI) technique by collision-induced dissociation on a Bruker APEX-4 (7 Tesla) instrument. The samples were dissolved in acetonitrile, diluted in spray solution (methanol/water 1:1 *v*/*v* + 0.1 M formic acid), and infused using a syringe pump with a flow rate of 2 μL/min. External calibration was conducted using Arginine cluster in a mass range *m*/*z* 175–871. Low-resolution mass spectra (LRMS) were measured by Applied Biosystems-MDS SCIEX API 3200 LC/MS/MS system (Applied Biosystems, Foster City, CA, USA), employing the positive mode using an electrospray ionizer (ESI) which was operated at 5.0–5.5 kV, with the capillary heater at 350 °C, sheath gas pressure 45 psi (USA), and an ion trap analyzer. Molecular weight was recorded as (AMU) + 1, as the positive mode of ESI adds 1 AMU to molecular ion peak. Some compounds were recorded as AMU + 2, as the positive mode of ESI adds 1 AMU to molecular ion peak and so does the iontrap analyzer.

#### Synthesis of Novel Title Compounds **4b** and **4c**

##### Synthesis of Compound **1**

Compound **1** was previously reported by our group [27]. It was synthesized following the same procedure with slight modification. Compounds **2**, **3** (**a**, **d** and **e**) were also prepared and previously reported, and their spectral data were reported therein [29]. Compounds **4**, **5** were prepared following a similar procedure by our lab [31]. Compounds **4**, **5** (**b**, **c**) were prepared for the first time and reported in this work for their activity, whereas compounds **4**, **5** (**a**, **d**, **e**) are also new and sent for publication for other activities [32].

##### Synthesis of New Compounds **4**, **5** (**b**, **c**) (Scheme 1)

*8-Amino-1-cyclopropyl-6-fluoro-7-[(4-methylphenyl)amino]-4-oxo-1,4-dihydroquinoline-3-carboxylic acid* (**4b**) (Scheme 1). A mixture of 1-cyclopropyl-6-fluoro-7-[(4-methylphenyl)amino]-8-nitro-4-oxo-1,4-dihydroquinoline-3-carboxylic acid (**3b**, 0.38 mmol, 0.15 g) in 4 mL of 12 N HCl, left stirring in ice bath (2–5 °C) for 15 min. After that, the ice bath was removed, and (0.38 g, 1.7 mmol) stannous chloride (SnCl_2_) was added portion wise and the reaction mixture left stirring overnight and monitored by TLC until completion. Then, the reaction mixture was poured on crushed ice to precipitate product that is collected by filtration and left to dry. Color of solid compound: yellow; yield ≈ 70.7% (0.098 g); M.p.: 205–210 °C (decomposition); *R*_f_ value in system 1 = 0.33. ^1^H-NMR (300 MHz, DMSO-*d*_6_): 1.20 (m, 4H, H2-2′, H2-3′), 2.20 (s, 3H, Ar-CH_3_), 4.52 (m, 1H, H-1′), 5.69 (br s, 2H, NH_2_), 6.59 (d, *J* = 7.9 Hz, 2H, H-2′′, H-6′′), 6.98 (d, *J* = 7.8 Hz, 2H, H-3′′, H-5′′), 7.35 (d, ^3^*J*_H-F_ = 9.9 Hz, 1H, H-5), 7.70 (br s, 1H, NH, exchangeable), 8.75 (s, 1H, H-2), 14.67 (br s, 1H, COOH). ^13^C-NMR (75 MHz, DMSO-*d*_6_): 10.61 (C-2′, C-3′), 20.61 (CH_3_), 39.91 (C-1′), 98.39 (d, ^2^*J*_C-F_ = 23 Hz, C-5), 106.60 (C-3), 114.86 (C-2′′, C-6′′), 115.09 (C-4′′), 121.25 (C-4a), 125.08 (C-8a), 127.86 (d, ^2^*J*_C-F_ = 19.5 Hz, C-7), 129.74 (C-3′′, C-5′′), 139.04 (C-8), 150.05 (d, ^1^*J*_C-F_ = 254 Hz, C-6), 151.08 (C-2), 158.53 (C-1′′), 166.30 (COOH), 172.21 (C-4). IR (KBr): ν 3371, 3093, 2924, 2430, 1712, 1612, 1512, 1450, 1327, 2288, 1180, 1087, 1026 cm^−1^. HRMS (ESI, +ve): calc. for C_20_H_18_FN_3_NaO_3_ [M + Na]^+^ (390.12299). Found 390.12244. Anal. Calcd. for C_20_H_16_FN_3_O_3_ (367.37): C, 65.39; H, 4.49; N, 11.44 Found C, 65.73; H, 4.82; N, 11.05.

*8-Amino-1-cyclopropyl-6-fluoro-7-(4-methoxy-phenylamino)-4-oxo-1,4-dihydro-quinoline-3-carboxylic acid* (**4c**) (Scheme 1). A mixture of (**3c**, 0.4 g, 1 mmol) in 4 mL of 12 N HCl was left stirring in ice bath (0–5 °C) for 15 min. After that, the ice bath was removed, and (0.75 g, 4 mmol) stannous chloride (SnCl_2_) was added portion wise. The reaction mixture left stirring overnight and was monitored by TLC until completion. Then, the reaction mixture was poured on crushed ice to precipitate a red product that is collected by filtration and left to dry. Yield = 0.34 g (≈61%). M.p.: 190–192 °C; *R*_f_ value in system 1 = 0.37. ^1^H-NMR (300 MHz, DMSO-*d*_6_): 1.04, 1.23 (2m, 4H, H2-2′, H2-3′), 3.73 (s, 3H, OCH_3_), 3.8 (m, 1H, NCH-1′), 4.15 (m, 2H, NH_2_), 7.07 (dd, *J*_o_ = 7.2 Hz, *J*_m_ = 1.8 Hz, 2H, H-2′′, H-6′′), 7.41 (dd, *J*_o_ = 9 Hz, *J*_m_ = 2.7 Hz, 2H, H-3′′, H-5′′), 7.65 (m, 1H, NH), 8.54 (d, ^3^*J*_H-F_ = 11.4 Hz, H-5), 8.91 (s, 1H, H-2), 14.46 (br s, 1H, COOH). ^13^C-NMR (75 MHz, DMSO-*d*_6_): 7.94 (2C, C-2′, C-3′), 39.74 (C-1′), 55.82 (OCH_3_), 98.53 (C-3), 109.85 (d, ^2^*J*_C-F_ = 22.5 Hz, C-5), 115.19 (d, ^3^*J*_C-F_ = 16.78 Hz, C-5), 115.9 (C-4a), 124.83 (2C, C-3′′, C-5′′), 125.2 (2C, C-2′′, C-6′′), 131.9 (C-8a), 132.53 (C-8), 140.82 (C-7), 147.2 (C-1′′), 148.03 (C-2), 149.4 (d, ^1^*J*_C-F_ = 240 Hz, C-6), 157.28 (C-4′′), 166.4 (COOH), 177.5 (C-4). IR (KBr): ν 3356, 2924, 2854, 2484, 2291, 1635, 1512, 1458, 1381, 1249, 1180, 1033 cm^−1^. HRMS (ESI, +ve): calc. for C_20_H_18_FN_3_NaO_4_ [M + H]^+^ (406.11790). Found 406.11736. LRMS (ES, +ve) *m*/*z* calc. for C_20_H_18_FN_3_O_4_ (383.13): Found 383.3 (100%), 378.4 (36%), 376.6 (5%), 370.4 (2%), 367.3 (39%), 364.6 (20%), 354.0 (95%), 346.4 (8%), 339.3 (11%), 336.1 (80%), 321.1 (5%), 310.6 (61%), 295.5 (12%), 258.1 (2%), 149.1 (1%), 130.3 (3%), 78.9 (1%), 73.6 (12%), 59.0 (37%), 57.2 9 (2%). Anal. Calcd. for C_20_H_18_FN_3_O_4_ (383.37): C, 62.66; H, 4.73; N, 10.96 Found C, 62.94; H, 4.98; N, 11.14.

*9-Cyclopropyl-4-fluoro-6-oxo-3-p-tolyl-6,9-dihydro-3H-*[1,2,3]*triazolo*[4,5-*h*]*quinolone-7-carboxylic acid* (**5b**) (Scheme 1). Compound **5b** was synthesized through cyclization of preceding reduced acid **4b**. The amino **4b** (**4b**, 0.6 g, 1.6 mmol) was treated with 20 mL aqueous HCl, then left stirring in ice bath (0–5 °C) for 15 min. NaNO_2_ (0.14 g, 1.6 mmol) dissolved in 10 mL H_2_O is added drop wise. The reaction mixture was left stirring overnight. Progress of cyclization reaction was monitored by TLC and was completed within 24 h. Thereafter, the reaction mixture was cooled, poured onto crushed ice (250 g), and the resulting off-white precipitate was collected, washed with cold water (2 × 20 mL), and left to dry. Yield = 0.45 g (≈67%). M.p.: 280–282 °C; *R*_f_ value in system 1 = 0.39. ^1^H-NMR (300 MHz, CDCl_3_): 1.25 (m, 2H, CH_2_-2′), 1.35 (m, 2H, CH_2_-3′), 2.44 (s, 3H, CH_3_), 4.62 (m, 1H, H-1′), 7.35 (d, *J* = 8 Hz, 2H, H-3′′, H-5′′), 7.68 (d, *J* = 8 Hz, 2, H-2′′, H-6′′), 8.24 (d, ^3^*J*_H-F_ = 18.25 Hz, 1H, H-5), 8.8 (s, 1H, H-8), 14.9 (br s, 1H, COOH). ^13^C-NMR (75 MHz, CDCl_3_): 9.96 (2C, C-2′, C-3′), 22.15 (CH_3_), 41.84 (C-1′), 108.56 (C-5), 114.85 (d, ^2^*J*_C-F_ = 22.13 Hz, C-5), 115.1 (2C, C-3′′, C-5′′), 122.4 (C-5a), 125.6 (C-1′′), 127.75 (C-2′′, C-6′′), 129.5 (C-3a), 130.6 (C-9a), 135.2 (C-9b), 140.3 (C-4′′), 151.4 (d, ^1^*J*_C-F_ = 248 Hz, C-4), 147.6 (C-8), 166.74 (COOH), 174.2 (C-6). IR (KBr): ν 3368, 3044, 2820, 2310, 1609, 1516, 1432, 1176, 1044, 960 cm^−1^. LRMS (ES, +ve) *m*/*z* calc. for C_20_H_15_FN_4_O_4_ (378.11): Found 379.4 (100%, M + 1), 362.4(15%), 334.4 (8%), 323.3 (10%), 310.1 (4%), 146.2 (4%), 118.4 (15%), 80.1 (2%), 54.2 (4%). Anal. Calcd. for C_20_H_15_FN_4_O_3_ (378.36): C, 63.49; H, 4.00; N, 14.81 Found C, 63.81; H, 3.74; N, 15.28.

*9-Cyclopropyl-4-fluoro-3-(4-methoxy-phenyl)-6-oxo-6,9-dihydro-3H-*[1,2,3]*triazolo*[4,5-*h*]*quinoline-7-carboxylic acid* (**5c**) (Scheme 1). Compound **5c** was synthesized through cyclization of preceding reduced acid **4c**. The amino acid (**4c**, 0.8 g, 2.1 mmol) was treated with 20 mL aqueous HCl, and then left stirring in ice bath (0–5 °C) for 15 min. NaNO_2_ (0.14 g, 2.1 mmol) dissolved in 10 mL H_2_O was added drop wise to the mixture. The reaction mixture was left stirring overnight. Progress of cyclization reaction was monitored by TLC and was completed within 20 h. Thereafter, the reaction mixture was cooled, poured onto crushed ice (250 g), and the resulting off-white precipitate was collected, washed with cold water (2 × 20 mL) and left to dry. Yield = 0.6 g (≈75%). M.p.: 280–282 °C; *R*_f_ value in system 1 = 0.36. ^1^H-NMR (300 MHz, CDCl_3_): 1.45 (m, 2H, CH_2_-2′), 1.56 (m, 2H, CH_2_-3′), 3.78 (s, 3H, OCH_3_), 4.59 (m, 1H, H-1′), 7.24 (d, *J* = 8.2 Hz, 2H, H-3′′, H-5′′), 7.8 (d, *J* = 8.2 Hz, 2H, H-2′′, H-6′′), 8.17 (d, ^3^*J*_H-F_ = 16.36 Hz, 1H, H-5), 8.92 (s, 1H, H-8), 15.2 (br s, 1H, COOH). ^13^C-NMR (75 MHz, CDCl_3_): 10.24 (2C, C-2′, C-3′), 41.84 (C-1′), 56.17 (OCH_3_), 109.56 (C-7), 114.85 (d, ^2^*J*_C-F_ = 22.13 Hz, C-5), 115.1 (2C, C-3′′, C-5′′), 122.4 (C-5a), 127.75 (C-2′′, C-6′′), 129.5 (C-3a), 130.6 (C-9a), 135.2 (C-9b), 140.6 (C-1′′), 146.7 (d, ^1^*J*_C-F_ = 258.52 Hz, C-4), 148.45 (C-8), 161.00 (C-4′′), 165.4 (COOH), 176.5 (C-6). IR (KBr): ν 3359, 3087, 2914, 2332, 1617, 1535, 1421, 1164, 1087, 1007 cm^−1^. LRMS (ES, +ve) *m*/*z* calc. for C_20_H_15_FN_4_O_4_ (394.11): Found 395.4 (100%, M + 1), 381.6 (6%), 377.3 (31%), 372.4 (1%), 361.4 (4%), 345.2 (1%), 333.4 (3%), 325.6 (9%), 317.4 (1%), 141.1 (1.2%), 122.4 (17%), 104.3 (2%), 83.3 (1%), 67.2 (1%), 59.2 (2%). Anal. Calcd. for C_20_H_15_FN_4_O_4_ (394.36): C, 66.91; H, 3.83; N, 14.21 Found C, 66.61; H, 4.07; N, 14.38.

### 3.2. Microbiological Methods and Anti-Microbial Assays

#### 3.2.1. Bacterial Strains and Growth Conditions

Twelve *H. pylori* clinical isolates and one reference strain of *H. pylori* (NCTC 11916). The clinical strains were isolated from gastric biopsy samples obtained by a gastroenterologist of the Jordan University Hospital during a routine endoscopy. The gastric biopsy material was processed according to the standard methodology [37]. Briefly, each biopsy for culture was homogenized using a tissue homogenizer (IKA, Staufen, Germany). Aliquots of 100 μL of the homogenate were primarily cultured on Columbia blood agar (Oxoid, Hampshire, UK) supplemented with 7% (*v*/*v*) horse blood and Dent selective supplement (Oxoid, Hampshire, UK). Subcultures of the bacteria were performed using the same plates but without the Dent supplement. All of the plates were incubated at 37 °C under microaerophilic conditions using CampyGen atmosphere generating system (Oxoid, Hampshire, UK) in anaerobic jars for 5–7 days. Growth of *H. pylori* was confirmed according to colony morphology, Gram staining, microaerophilic growth (at 37 °C), biochemical tests (positive for oxidase, catalase and urease), and subsequently by standard PCR of 16S ribosomal DNA test [38]. *H. pylori* cultures were stored at −70 °C in Trypticase soy broth (Oxoid, Hampshire, UK) containing 10% (*v*/*v*) fetal calf serum (PAA, Pasching, Austria) and 15% glycerol.

#### 3.2.2. Antimicrobial Susceptibility Testing and Minimal Inhibitory Concentration Determination

Bacterial suspensions were prepared to the 2 McFar← land’s standard and subsequently uniformly spread on a solid growth medium in a Petri dish. Sterile paper discs (6 mm in diameter; Oxoid, Hampshire, UK) were impregnated with 25 μL of the each compound, and were placed on the surface of each agar plate. Plates were incubated for 5–7 days under appropriate cultivation conditions. Antibacterial activity was determined by the production of an inhibition zone around the impregnated disc with the compounds. Disks impregnated with DMSO served as negative controls, and disks with standard antibiotics (ciprofloxacin, and metronidazole, Oxoid, Hampshire, UK) served as positive controls. The minimal inhibitory concentrations (MICs) of each compound were determined by the two-fold agar dilution method as previously described [39]. In brief, each compound was serially diluted in DMSO and incorporated to molten Columbia blood agar plates supplemented with 7% (*v*/*v*) horse blood. Spot of *H. pylori* (1 × 10^5^ CFU) was applied on the surface of each plate, and the growth of visible colonies was determined after seven days of incubation at 37 °C under microaerophilic conditions. MIC was recorded as the lowest concentration that inhibited visible growth of organisms; and the plates with the standard antibiotics served as positive controls, and plates with DMSO served as negative controls. Triplicates of each tested compound were performed, and the average of the results was taken.

#### 3.2.3. Determination of In Vitro Interaction

Antimicrobial interactions between each compound and metronidazole against three strains (two clinical isolates and one reference strain) of *H. pylori* were evaluated by the standard checkerboard titration method [40]. Each compound and metronidazole was dissolved in DMSO and distilled water, respectively. The bacterial inoculum, media, and culture conditions were the same as those described for the MIC determination mentioned above. Experiments were performed in triplicate.

The fractional inhibitory concentrations (FICs) were calculated as follows:

FIC = (MIC of drug A in combination/MIC of drug A alone) +‏ (MIC of drug B in combination/MIC of drug B alone)

The FIC indices were interpreted as follows: ≤0.5, synergy; 0.5–1, additive; 0.1–4.0, indifference; >4.0, antagonism.

### 3.3. Urease Inhibition Assay

Urease inhibition was performed as described elsewhere [41]. Briefly, 10 μL of 10^8^ CFU/mL *H. pylori* suspension was incubated with equal amount of serially diluted compound in 96 well microplates for 30 min at 37 °C. Subsequently, 200 μL of detection reagent composed of 50 mM phosphate buffer, pH 6.8 containing 500 mM urea, and 0.02% phenol red was added to each well. The color development was measured at 555 nm in 5 min intervals. Controls included bacteria with the reagent and reagent with each compound. The percentage of inhibition was calculated by the equation % inhibition = [(activity without compound − activity with compound)/(activity without compound)] × 100. The activity was compared to a reference urease inhibitor acetohdyroxamic acid (Sigma Aldrich, St. Louis, MO, USA).

## 4. Conclusions

This study was successfully implemented and introduced new 8-amino fluoroquinolone derivatives using a new procedure developed within the course of this study. The fluoroquinolone derivatives were fully identified and characterized using NMR, IR, electrochemical analysis (EA), and MS. Moreover, all of these new compounds have interesting antimicrobial activity against metronidazole-resistant *H. pylori*. Losing synergistic activity between new derivatives and metronidazole might be associated with increased lipophilicity of the new complex due to inter-molecular hydrogen bond of the 8-amino group in these compounds and metronidazole. With the emergence of *H. pylori* resistance to a number of antimicrobial agents (especially metronidazole), the bacteria is becoming a major health problem and there is a need to develop novel agents to treat and eradicate *H. pylori* infections. The results of this study suggest that all of the newer 8-amino 7-substituted fluoroquinolone and their correspondent cyclized triazolo derivatives (with their good to excellent activities) may have potential to be used as anti-*H. pylori*, and this should be explored.

## Figures and Tables

**Table 1:** Zones of inhibition (mm) of synthesized compounds (1 mg/mL), ciprofloxacin, and metronidazole against clinical strains of *H. pylori* (1–12) and control strain.

Tested Compounds	Zones of Inhibition (mm)												
	Clinical Isolates												Control Strain
	1	2	3	4	5	6	7	8	9	10	11	12	
**4a**	25	45	35	40	46	43	40	16	11	38	41	42	40
**4b**	25	46	38	44	48	45	42	21	15	42	40	44	49
**4c**	24	38	34	37	35	38	35	20	14	29	37	35	45
**4d**	15	35	30	35	38	36	44	18	20	25	35	40	44
**4e**	15	35	34	40	45	42	43	17	12	30	38	36	43
**5b**	10	15	12	10	20	12	13	15	7	17	18	16	21
**5c**	16	30	25	28	29	30	22	15	7	25	29	28	35
**5d**	22	22	20	19	24	20	16	14	8	20	22	20	32
**5e**	20	25	15	15	24	17	14	13	7	19	24	22	25
CIP	20	60	0	50	53	65	59	0	0	64	50	55	65
MTZ	10	0	0	0	0	10	0	0	0	0	0	0	0
DMSO	0	0	0	0	0	0	0	0	0	0	0	0	0

CIP: ciprofloxacin; MTZ: metronidazole.

**Table 2:** Minimum inhibitory concentration (MIC, μg/mL) of tested compounds and metronidazole against clinical strains of *H. pylori* (1–12) and control strain by two-fold agar dilution method.

Compounds	Minimum Inhibitory Concentration (μg/mL)												
	Strain Number												Control Strain
	1	2	3	4	5	6	7	8	9	10	11	12	
**4a**	2.5	0.6	2.5	2.5	1.3	0.6	1.3	5	5	2.5	0.6	1.3	1.3
**4b**	0.6	1.3	2.5	2.5	0.6	1.3	0.6	1.3	0.6	2.5	0.6	0.6	1.3
**4c**	5	2.5	5	5	2.5	2.5	1.3	2.5	5	5	0.6	0.6	1.3
**4d**	2.5	5	1.3	2.5	2.5	1.3	5	5	2.5	1.3	1.3	1.3	1.3
**4e**	5	2.5	5	5	5	2.5	5	2.5	2.5	5	2.5	2.5	2.5
**5b**	2.5	5	1.3	1.3	2.5	1.3	2.5	5	5	1.3	1.3	1.3	5
**5c**	5	1.3	1.3	2.5	2.5	1.3	1.3	5	10	2.5	1.3	1.3	2.5
**5d**	2.5	1.3	1.3	1.3	2.5	1.3	2.5	5	10	2.5	1.3	1.3	2.5
**5e**	5	2.5	2.5	5	2.5	1.3	5	10	10	2.5	1.3	1.3	2.5
CIP	0.3	0.6	0.3	0.6	0.3	0.04	0.6	0.04	0.08	0.04	0.6	0.6	0.6
MTZ	64	64	64	32	128	64	128	32	64	64	128	64	128

**Table 3:** Fractional inhibitory concentration (FIC) values of tested compounds in combination with metronidazole against clinical strains of *H. pylori* (11, 12) and control strain by standard checkerboard titration method with two-fold serial agar dilutions.

Compounds	FIC Values (Index) (MIC Combination)			FIC Mean
	Strain Number			
	11	12	Control Strain	
**4a-MTZ**	2.039 (=)	0.519 (+)	0.519 (+)	1.025 (=)
**4b-MTZ**	2.039 (=)	1.019 (=)	0.519 (+)	1.192 (=)
**4c-MTZ**	4.078 (−)	2.039 (=)	0.519 (+)	2.212 (=)
**4d-MTZ**	1.039 (=)	0.519 (+)	0.519 (+)	0.692 (+)
**4e-MTZ**	1.078 (=)	0.539 (+)	0.539 (+)	0.718 (+)
**5b-MTZ**	0.259 (*)	0.519 (+)	0.072 (*)	0.283 (*)
**5c-MTZ**	0.519 (+)	0.259 (*)	0.269 (*)	0.349 (*)
**5d-MTZ**	0.259 (*)	0.519 (+)	0.269 (*)	0.349 (*)
**5e-MTZ**	0.519 (+)	0.259 (*)	0.134 (*)	0.304 (*)

(*): Synergism. (+): Additive. (=): Indifferent. (−): Antagonism.
